# Timing of Cervico-Vaginal Cytokine Collection during Pregnancy and Preterm Birth: A Comparative Analysis in the PRINCESA Cohort

**DOI:** 10.3390/ijerph18073436

**Published:** 2021-03-26

**Authors:** Miatta A. Buxton, Noemi Meraz-Cruz, Brisa N. Sanchez, Betsy Foxman, Marisol Castillo-Castrejon, Marie S. O’Neill, Felipe Vadillo-Ortega

**Affiliations:** 1 Department of Epidemiology, School of Public Health, University of Michigan, Ann Arbor, MI 48109, USA; bfoxman@umich.edu (B.F.); marieo@umich.edu (M.S.O.); 2 Unidad de Vinculación Científica de la Facultad de Medicina, Universidad Nacional Autónoma de México en el Instituto Nacional de Medicina Genómica, Mexico City 14610, Mexico; noemi_meraz@hotmail.com (N.M.-C.); felipe.vadillo@gmail.com (F.V.-O.); 3 Department of Epidemiology and Biostatistics, School of Public Health, Drexel University, Philadelphia, PA 19104, USA; bns48@drexel.edu; 4 Stephenson Cancer Center, Harold Hamm Diabetes Center, University of Oklahoma Health Sciences Center, Oklahoma City, OK 73104, USA; Marisol-CastilloCastrejon@ouhsc.edu; 5 Department of Environmental Sciences, School of Public Health, University of Michigan, Ann Arbor, MI 48109, USA

**Keywords:** longitudinal data, inflammation, preterm birth, comparative analysis, timing of sample collection during pregnancy

## Abstract

Preterm birth (PTB), defined as birth before 37 completed weeks of gestation, is a major cause of infant morbidity and mortality. Inflammation is an important component in the physiopathologic pathway leading to PTB but results from cross-sectional studies on associations between inflammation, as measured by cytokines, and PTB are inconsistent. Timing of cytokine measurement during pregnancy varies between studies and may contribute to inconsistent findings. We investigated the effects of timing on associations between 16 cervico-vaginal cytokines (Eotaxin, IL-10, IL-12p40, IL-17, IL-1RA, sIL-2rα, IL-1a, IL-1β, IL-2, IL-6, IP-10, MCP-1, MIP-1α, MIP-1β, TNFα, and VEGF) and PTB among 90 women throughout pregnancy. We used logistic regression to compare associations between concentrations of cervico-vaginal cytokines from periods in pregnancy and PTB. Trimester 1 cytokines had the strongest positive associations with PTB; for example, OR = 1.76 (95% confidence interval: 1.28, 2.42) for IL-6. Second and third trimester associations were weaker but largely positive. IL-1α was the only cytokine with a negative association (trimesters 2, 3 and overall pregnancy). Strong first trimester associations between cytokines and PTB suggest that measuring cytokines early in pregnancy may hold promise for early identification of PTB risk. Variations in cytokine measurement during pregnancy may contribute to inconsistencies among studies.

## 1. Introduction

Preterm birth (PTB, birth < 37 weeks gestation) is a major public health problem worldwide [1,2]. The burden of preterm birth varies by setting, with higher rates and worse outcomes in low-income countries [1]. A number of risk factors—including behavioral, medical and socio-demographic and environmental factors—are associated with preterm birth [3]. However, interventions targeting some of these risk factors such as prophylactic and medically indicated antibiotic treatments have had mixed results [4,5,6,7,8,9,10]. Consequently, much remains to be understood regarding how these risk factors work to influence preterm risk, despite decades of research [11]. Understanding the underlying mechanisms and pathways is crucial for potentially developing PTB screening and prevention programs as alternative avenues to pursue in the fight against preterm birth.

The inflammatory response, involving fetal membranes, decidua and cervix, is one of the pathways linked to the development of PTB [3,12,13,14]. The physiological adaptations associated with pregnancy can moderate changes in cytokine expression [12], but cytokines are also associated with pathologic processes and may potentially predict PTB [15,16,17]. However, available evidence on the association between cytokines and PTB is inconsistent, perhaps because most published studies measured cytokines at a single point during pregnancy, and vary in cytokines measured, the stage of pregnancy when samples were obtained, samples assayed and methodologies for cytokine measurement. For example, a prospective cohort study of 218 women found that women with high anti-inflammatory/low pro-inflammatory first trimester cervical cytokines were at higher risk of early spontaneous preterm birth compared to women who had low anti-inflammatory/high pro-inflammatory or balanced levels [18]. Similarly, a prospective pilot study conducted among 39 women found that levels of pro-inflammatory cytokine IL-6 obtained from vaginal samples were lower in women who delivered preterm compared to women who delivered full term [19]. By contrast, a nested case–control study conducted among 250 participants reported higher mean concentrations of IL-6 in women who delivered preterm compared to term [20]. Similar inconsistencies have been noted in studies using serum/plasma samples [21,22,23].

Our study addresses these previous limitations and uses a design that includes the same set of participants across the study. We utilized 16 cytokines obtained from monthly cervico-vaginal samples to estimate the associations between cytokines from different points in pregnancy and PTB.

## 2. Materials and Methods

### 2.1. Study Participants

We analyzed data from the Pregnancy Research on Inflammation, Nutrition, & City Environment: Systematic Analyses (PRINCESA) cohort, a longitudinal study (enrollment 2009–2014) based in Mexico City [24]. The research study received approval from the University of Michigan Institutional Review Board, and the ethics committees from the Secretaría de Salud del Gobierno de la Ciudad de México (Mexico City) and the School of Medicine of the National Autonomous University of Mexico.

Study participants were pregnant women in Mexico City, Mexico. Inclusion criteria included singleton pregnancies, and resided and/or worked in Mexico City and surrounding areas. In addition, participants had to be 18 years or older, in their first or second trimester of pregnancy, and able to attend approximately monthly prenatal visits. Exclusion criteria included presence of medical or obstetric complications in the current pregnancy. All participants provided written informed consent. Clinical samples, behavioral, and demographic information were collected monthly. The parent study included 935 participants and birth outcomes were available for 838 participants. This analysis was limited to the 90 participants who met all of the following requirements; (i) participants had to have had at least three visits (ii) at least one visit during each trimester (iii) cytokine measures for each corresponding visit, and birth outcome including gestational age had to be available for each participant. There were no fetal abnormalities among participants in the current study. Participants who developed obstetric complications were referred to a tertiary level hospital and did not continue follow up in the study.

### 2.2. Preterm Birth Definition

Fetal gestational age was calculated using the date of visit and the reported first day of the last menstrual period (LMP) and confirmed by ultrasound; gestational age at birth was calculated based on the infant’s date of birth and the first day of the LMP. Both calculations were based on reliable and accurate recall of LMP from data collected during screening. PTB was defined as being born before 37 weeks of gestation. Spontaneous preterm labor and pre-labor rupture of the membranes were included.

### 2.3. Collection of Biological Samples

Sampling occurred by the use of a vaginal mirror rinsed in a sterile saline solution to visualize the cul de sac. Cervico-vaginal samples were obtained using a Dacron swab rotated for 10 s in the cervico-vaginal section of the reproductive tract. Collected samples were placed in a buffering solution and transported to the laboratory. Two additional samples of the exudate were taken using a similar method and were processed for conventional microbiologic cultivation and for confirmatory probes if infection was suspected. Samples were collected during each visit and frozen at −20 °C for later processing. Samples used in this study were collected between 2010 and 2014 and cytokines were quantified between 2011 and 2014.

### 2.4. Quantification of Cytokines

The following cytokines were quantified in picograms/milliliter (pg/mL) from cervico-vaginal samples: Eotaxin, Interferon gamma (IFNγ), Interleukin (IL)-10, IL-12p40, IL-12p70, IL-17, IL-1RA, soluble IL-2 receptor alpha (sIL-2rα), IL-1α, IL-1β, IL-2, IL-4,IL-6, IL-8, Interferon gamma inducible protein (IP-10), monocyte chemoattractant protein-1 (MCP-1), macrophage inflammatory protein 1 alpha (MIP-1α), macrophage inflammatory protein-1 beta (MIP-1β), tumor necrosis factor alpha (TNFα), and vascular endothelial growth factor (VEGF). Cytokine quantification was done using the Millipore MILLIPLEX^®^ MAP human cytokine/chemokine magnetic bead panel kit (Millipore Corporation, Billerica, MA, USA) based on the manufacturer’s published protocol. Analyses were performed on 50 µL of previously frozen duplicate cervico-vaginal exudate samples. Cytokine data included left and right censored observations. Limit of detection (LOD)/√2 (left censored) and a value of 10,010 (right censored) were substituted for these observations.

### 2.5. Statistical Analysis

Cytokine distributions were evaluated using histograms to assess whether cytokine data needed to be transformed. Fisher’s exact test and T-test were used to evaluate differences between term and preterm births. To evaluate differences in the distribution of cytokine concentrations between groups defined by preterm birth status, we used the non-parametric Kruskal–Wallis test because the normality of residuals assumption required to use analysis of variance was not met. We evaluated the association between each cytokine and PTB on the same group of participants over time. To account for potential cytokine variability within each trimester and avoid having single measures represent cytokine levels during a trimester, we averaged individual cytokine measurements for each exposure period (trimester-specific and overall pregnancy). Comparative analyses were conducted using logistic regression models for individual cytokines for the following averages: i. first trimester; ii. second trimester; iii. third trimester; and iv. overall pregnancy. *p*-values from the logistic regression models were adjusted using Benjamini–Hochberg corrections for multiple testing [25]. All analyses were conducted using SAS 9.4 (SAS Institute, Cary, NC, USA).

## 3. Results

A group of ninety participants (78 term and 12 preterm births) was included in study. The median gestational age at enrollment was higher among term births than preterm births (11.2 weeks vs. 10.3 weeks). Other than finding that participants who delivered preterm babies were more likely to be nulliparous (*p* < 0.05), we observed no statistically significant associations between demographic characteristics and preterm birth status (Table 1).

Results presented are limited to the 16 cytokines where at least 50% of measurements were above the LOD. Thus, IL-4, IL-8, INFγ, and IL-12p70 were excluded from further analysis. The percent below the LOD for the included cytokines varied and ranged from 2.5–37.9% across cytokines. Mean ranks from Kruskal Wallis tests were significantly different for eight (IL-1β, IL-1RA, IL-2, IL-6, IP-10, MCP-1, MIP-1α, and VEGF), seven (IL-1β, IL-1RA, IL-2, IL-6, IP-10, MIP-1α, and VEGF), two (IL-1β and IL-6) and nine(IL-1β, IL-1RA, IL-2, IL-6, IP-10, MCP-1, MIP-1α, TNFα, and VEGF) cytokines from the first, second, third trimesters and overall pregnancy average, respectively. Cytokines IL-6 and IL-1β were the two cytokines that were significantly different across all points in pregnancy between term births and preterm births (Table 2).

Individual cytokines from the first trimester had the strongest positive associations with PTB; 14 cytokines (Eotaxin, IL-10, IL-12p40, IL-17, sIL-2rα, IL-1β, IL-2, IL-6, IP-10, MCP-1, MIP-1α, MIP-1β, TNFα, and VEGF) were either statistically significant or marginally significant after adjusting for multiple testing. Models using entire pregnancy average for each cytokine had 11 estimates that were either statistically or marginally significant compared to eight for second trimester concentrations. First trimester VEGF concentrations had the strongest association with PTB (odds ratio = 2.11; 95% CI 1.43, 3.10) and remained the strongest statistically significant predictor at other points during pregnancy. In general, statistically significant associations were fewer and the strength of the associations was attenuated as the pregnancy progressed. IL-1α was the only cytokine that exhibited a protective association (Table 3).

## 4. Discussion

We compared the distributions of 16 cervico-vaginal cytokines at each trimester of pregnancy between women who delivered term or preterm babies, and examined the age-adjusted associations between cytokine concentrations and risk of preterm birth. Samples across trimesters were obtained from the same group of women, a key strength of this longitudinal design. Both significant differences in cytokine concentrations and age-adjusted associations were found, with the strength of associations varying across trimesters. Cytokines obtained during the first trimester were the strongest and most frequent predictors of PTB. This finding suggests that processes occurring early on in pregnancy are potentially useful in identifying women at risk of delivering before 37 completed weeks of gestation and may inform future studies directed at early prevention of preterm labor. During pregnancy, the feto-maternal tolerance has to be established to allow growth and development of the fetus, while concurrently activating the innate and adaptive mechanisms to promote survival of the developing fetus. The resulting immune mechanisms involved in these processes may be systemic or operate in the local components of the maternal-fetal unit [26]. Feto-maternal tolerance is established in the first trimester, so any alteration during this period can lead to a deficient immunotolerance or immune maladaptation. This may involve inflammatory responses at the maternal-fetal interface that could be involved in adverse pregnancy outcomes [27]. Therefore, it is plausible that cytokines measured during the first trimester are a better predictor of PTB because key physiological processes occur or are initiated during this period. Moreover, the first trimester is when the spiral artery, which is vital to the placenta and developing fetus, is being remodeled [28]. Disruption in the remodeling process may lead to placental under-perfusion once maternal blood flow is established and has been associated with complications and adverse pregnancy outcomes [28]. Furthermore, regulatory mechanisms are needed to balance inflammation to ensure development of the fetus and success of pregnancy [29]. Differences in the distribution, specifically the medians, of a number of cytokines between term and preterm births in the current study may indicate an imbalance in inflammation. A study conducted among 218 women with cervico-vaginal samples collected during the first trimester found that unbalanced inflammation (those with high anti-inflammatory/low pro-inflammatory cytokine levels) were at increased risks of preterm birth, odds ratio 7.7 (95% CI, 4.9–9.1) compared to those who had low anti-inflammatory/high pro-inflammatory cytokine levels or balanced inflammation [18]. Advancements in our understanding of what constitutes balanced versus unbalanced inflammation may play a crucial role in identifying persons at increased risks of preterm birth and should be investigated in future studies.

Identifying individuals who are at high risk of preterm birth during the first trimester may lead clinicians to closely monitor high-risk patients and provide an opportunity to treat risk factors, for example asymptomatic lower reproductive tract infection, associated with increased risks of preterm birth. In addition, in other cases where increased risks are not associated with lower reproductive tract infections, initiation of a low-dose aspirin regimen during pregnancy could play a role in reducing preterm birth risk and should be explored as a prevention for preterm birth. A recently published double-masked, placebo-controlled randomized trial of 11,976 participants conducted in six countries found a statistically significant reduction in preterm birth and other key measures among women taking low-dose aspirin initiated between the 6th week and end of the 13th week of pregnancy compared to women taking placebo [30]. Additionally, the use of a low-dose aspirin regimen is currently recommended by the United States Preventive Services Task Force for the prevention of morbidity and mortality among women at high risk for pre-eclampsia [31]; thus the use of low-dose aspirin during pregnancy is already established in obstetric care. Finally, preterm birth has a global impact with the highest burden in low-income countries [1], particularly in rural areas that may offer limited access to skilled clinicians and adequately equipped facilities [32]. In these areas, a significant percent of deliveries occur in facilities that cannot handle any obstetric or pediatric events outside of a normal natural delivery. Therefore, identifying high-risk individuals early during pregnancy may also reduce the impact (morbidity and mortality) associated with preterm birth if individuals are moved to facilities that could improve survival of both mother and child [33].

Several previous studies have evaluated associations between PTB and a limited number of cytokines obtained from a single point during pregnancy. Although some of these studies found associations between cytokine levels and PTB, results have been inconsistent, ranging from null, to positive and negative associations [11,19,20,34]. The reason for these inconsistencies is not entirely clear, but has been attributed to factors such as biomarker selection, and sampling timeframe [11] and may additionally include differences in biological compartments from which samples are taken to quantify cytokines. Although only cervico-vaginal samples were evaluated in the current study, a study conducted among 104 term births in this cohort (PRINCESA study) evaluated the association between exposure to two air pollutants (particulate matter less than 10 μm in aerodynamic diameter (PM_10_) and carbon monoxide (CO)) and the same cytokines (N = 7) quantified from cervico-vaginal samples and serum samples [35]. Using a number of comparisons, including Spearman correlations and intraclass correlation coefficients, and regression models, that study found differences in cytokine levels quantified from cervico-vaginal samples and serum samples. The authors concluded that the findings suggest that the immunologic responses may be compartment specific, which may contribute to inconsistencies found across studies using serum and cervico-vaginal samples and evaluating the association between cytokines and preterm birth. Cervico-vaginal and serum cytokines may need to be regarded as separate risk factors for preterm birth and future studies should evaluate how the underlying mechanisms differ.

Based on the limitations of previous studies, the simultaneous evaluation of 16 cervico-vaginal cytokines obtained over the course of gestation among a fixed group of women who delivered at term and preterm is a main strength of this study. Limitations of this work include that almost all participants were enrolled at three months of gestation, so we could not evaluate associations between cytokine levels at month 1 and 2 of gestation and PTB. Second, our sample size was relatively small, limiting our ability to adjust for known confounders other than maternal age. Third, we used substitution methods for censored cytokine observations and limitations have been reported when the percent above the LOD exceeds 25% [36]. The percent below the LOD ranged from 2.5–37.9% across cytokines in this study.

## 5. Conclusions

Positive associations between PTB and cytokines obtained during the first trimester suggest that samples taken early in pregnancy may be useful for risk screening. Consistently weaker associations seen later in pregnancy suggest that sampling from different periods of pregnancy may contribute to inconsistencies found in the PTB literature. Similarly designed studies are needed to further evaluate these findings.

## Figures and Tables

**Table 1:** Demographic and obstetric characteristics of Mexican women who delivered term (N = 78) and preterm babies (N = 12), Mexico City, PRINCESA Cohort 2009–2014.

Age	Term N (%)	Preterm N (%)
<20	13 (16.7)	4 (33.3)
20–35	52 (66.7)	8 (66.7)
>35	13 (16.7)	
**Pre-Pregnancy BMI**		
<18.5 kg/m^2^	2 (2.6)	
18.5–24.9 kg/m^2^	23 (29.5)	6 (50.0)
25–29.9 kg/m^2^	27 (34.6)	3 (25.0)
≥30 kg/m^2^	11 (14.1)	1 (8.3)
Missing	15 (19.2)	2 (16.7)
**Parity ^†^**		
Nulliparous	19 (24.4)	8 (66.7)
Parous	45 (57.7)	4 (33.3)
Missing	14 (18.0)	

^†^ Fisher’s exact test < 0.05.

**Table 2:** Median cytokine concentrations (pg/mL) by preterm birth status for the first, second and third trimesters and entire pregnancy average from the PRINCESA Cohort in Mexico City, 2009-14 (N = 90).

	Period-Specific Median Cytokine Concentrations (pg/mL) by Preterm Birth Status							
	First Trimester		Second Trimester		Third Trimester		Entire Pregnancy Average	
Cytokine	Term *n* = 78	Preterm *n* = 12	Term *n* = 78	Preterm *n* = 12	Term *n* = 78	Preterm *n* = 12	Term *n* = 78	Preterm *n* = 12
**Eotaxin**	17.10	16.35	19.25	20.40	20.78	14.95	20.23	16.92
**IL-10**	5.18	8.73	7.41	11.34	6.67	4.44	9.66	12.71
**IL-12p40**	9.16	14.13	14.51	19.73	11.93	14.98	14.29	13.68
**IL-17**	1.41	3.20	2.61	3.39	2.72	3.20	2.62	2.74
**IL-1α**	1393.75	369.68	1256.27	835.67	952.12	219.07	1312.24	465.72
**IL-1β**	104.88	3564.54 *	156.44	5126.72 *	66.67	4211.86 *	251.47	4225.49 *
**IL-1RA**	4350.24	10,010 *	5322.43	9820.62 *	5488.41	10010	5134.66	8530.72 *
**IL-2**	3.19	6.82 *	5.40	11.85 *	4.97	6.22	6.24	10.71 *
**IL-6**	11.29	137.79 *	12.77	31.21 *	10.25	33.11 *	14.43	90.06 *
**IP-10**	175.92	10,010 *	346.96	3425.53 *	193.19	2721.67	398.47	5081.50 *
**MCP-1**	99.96	3491.90 *	163.90	2939.19	161.12	1372.93	191.06	3460.61 *
**MIP-1α**	12.30	66.42 *	15.65	44.99 *	8.69	41.80	19.96	891.88 *
**MIP-1β**	25.90	44.48	28.70	40.30	28.16	41.44	38.98	72.95
**sIL-2Rα**	14.84	24.65	26.18	29.05	26.98	18.80	26.95	28.46
**TNFα**	2.75	2.81	3.09	4.22	3.17	4.44	3.87	75.06 *
**VEGF**	186.15	10,010 *	140.31	6706.56 *	128.49	186.47	155.46	4575.92 *

* *p* ≤ 0.05 from Kruskal Wallis Test LOD/√2 and 10,010 substituted left and right censored observations, respectively.

**Table 3:** Adjusted ^a^ odds ratios(OR) (95% confidence intervals) for preterm birth per log unit increase in cervico-vaginal cytokine concentrations (pg/mL) from models ^b^ including single trimester average, and entire pregnancy average, from the PRINCESA Cohort in Mexico City, 2009-14 (N = 90).

	Trimester-Specific Estimates OR (95% CI)			Entire Pregnancy Average OR (95% CI)
Cytokine	First Trimester	Second Trimester	Third Trimester	
**Eotaxin**	1.40 (1.05, 1.87) *	1.30 (0.99, 1.71)	1.18 (0.88, 1.57)	1.30 (0.97, 1.74)
**IL-10**	1.41 (1.07, 1.84) *	1.34 (1.02, 1.74) *	1.21 (0.91, 1.62)	1.37 (1.03, 1.83) *
**IL-12p40**	1.40 (1.06, 1.84) *	1.35 (1.03, 1.76) *	1.25 (0.96, 1.62)	1.35 (1.02, 1.78) *
**IL-17**	1.39 (1.04, 1.86) *	1.30 (0.98, 1.74)	1.35 (0.99, 1.85)	1.38 (1.01, 1.89)
**IL-1α**	0.79 (0.61, 1.02)	0.72 (0.54, 0.97) *	0.72 (0.54, 0.95) *	0.69 (0.51, 0.94) *
**IL-1β**	1.44 (1.07, 1.92) *	1.35 (1.03, 1.76) *	1.26 (0.99, 1.60)	1.40 (1.05, 1.87) *
**IL-1RA**	1.02 (0.79, 1.32)	1.10 (0.81, 1.49)	1.01 (0.79, 1.29)	1.05 (0.78, 1.41)
**IL-2**	1.59 (1.14, 2.21) *	1.55 (1.13, 2.13) *	1.40 (1.01, 1.94)	1.61 (1.14, 2.29) *
**IL-6**	1.76 (1.28, 2.42) ^†^	1.32 (0.97, 1.78)	1.43 (1.11, 1.83) *	1.53 (1.13, 2.07) *
**IP-10**	1.68 (1.19, 2.38) ^†^	1.62 (1.12, 2.34) *	1.35 (0.96, 1.88)	1.66 (1.13, 2.43) *
**MCP-1**	1.62 (1.17, 2.24) ^†^	1.31 (0.94, 1.82)	1.26 (0.94, 1.70)	1.45 (1.02, 2.06) *
**MIP-1α**	1.63 (1.23, 2.16) ^†^	1.41 (1.09, 1.83) *	1.27 (1.01, 1.61)	1.47 (1.12, 1.92) *
**MIP-1β**	1.43 (1.05, 1.96) *	1.28 (0.96, 1.70)	1.20 (0.90, 1.60)	1.32 (0.98, 1.78)
**sIL-2Rα**	1.39 (1.05, 1.85) *	1.32 (0.97, 1.79)	1.22 (0.89, 1.66)	1.34 (0.97, 1.84)
**TNFα**	1.34 (1.04, 1.73) *	1.32 (1.01, 1.72)	1.28 (0.97, 1.68)	1.38 (1.04, 1.84) *
**VEGF**	2.11 (1.43, 3.10) ^†^	1.83 (1.29, 2.59) ^†^	1.49 (1.07, 2.09) *	1.95 (1.34, 2.84) ^†^

^a^ Models adjusted for maternal age; ^b^ Cytokine measures up to eight months were included in the models; ^†^ *p* ≤ 0.05 after False Discovery Rate (FDR) adjustment for multiple testing; * *p* ≤ 0.1 after FDR adjustment for multiple testing LOD/√2 and 10,010 substituted left and right censored observations, respectively

## Data Availability

Not applicable.
